# A Novel Constrained Topographic Independent Component Analysis for Separation of Epileptic Seizure Signals

**DOI:** 10.1155/2007/21315

**Published:** 2007-08-06

**Authors:** Min Jing, Saeid Sanei

**Affiliations:** Centre of Digital Signal Processing, Cardiff University, Cardiff CF24 3AA, Wales, UK

## Abstract

Blind separation of the electroencephalogram signals (EEGs) using topographic independent component analysis (TICA) is an effective tool to group the geometrically nearby source signals. The TICA algorithm further improves the results if the desired signal sources have particular properties which can be exploited in the separation process as constraints. Here, the spatial-frequency information of the seizure signals is used to design a constrained TICA for the separation of epileptic seizure signal sources from the multichannel EEGs. The performance is compared with those from the TICA and other conventional ICA algorithms. The superiority of the new constrained TICA has been validated in terms of signal-to-interference ratio and correlation measurement.

## 1. INTRODUCTION

Epilepsy is the most common brain disorder only second to stroke, which affects nearly 60 million people in the world [1]. Many studies have been carried out from different aspects in order to explore the mechanisms of epileptogenesis and the possible solutions for anticipation and therapia [1–5]. Seizure detection has been under research for approximately three decades [6]. The most popular methods are based on time-frequency analysis [7] and artificial neural networks [8]. These methods do not exploit the multichannel electroencephalogram (EEG) information effectively.

Independent component analysis (ICA) has been increasingly applied to brain signal analysis for decomposition of multivariate EEGs to extract the desired sources. It has found a fruitful application in the analysis of multichannel EEGs [9] including epileptic seizure signals. The applications include the implementation of joint approximate diagonalization of eigenmatrices (JADE) and fastICA for seizure detection [10, 11], artifact rejection from epileptic intracranial EEGs by minimization of mutual information [12] and spatial filtering [13], and tracking of the epileptiform activity by incorporating the spatial constraint within the fastICA [14]. A novel approach proposed in [15, 16] applied an ICA approach to separate the seizure signals for prediction purpose and verified the predictability of epileptic seizure from the scalp EEGs. The main concept of this approach is to consider the seizures as independent components which are linearly and instantaneously combined together and with the noise and artifacts over the scalp. Subject to the mutual independency of the sources, the independent components can be separated by ICA algorithms and the seizure sources can be selected by postprocessing. The traditional nonlinear analysis methods can be applied to these seizure components for investigation of predictability. This approach can be further improved if a better performance of separation can be achieved. The objective of this work is to develop such method which can provide more plausible estimation of the seizure sources and eventually pave the way for the prediction of epileptic seizures from the scalp EEGs.

The conventional ICA model is built based on the statistical assumptions such that (1) the source signals are statistically independent; (2) the independent components must have nonGaussian distributions; (3) the number of independent components are less or equal to the number of input channels [17]. The ICA model has its own limitations. Apart from the scale ambiguity and the permutation problem, sometime the classic ICA cannot take all the prior physiological information into account and the results of separation cannot be interpreted physiologically. That is why in real applications the ICA algorithms have been modified to incorporate the relevant additional information into the separation processing as constraints to enhance both efficiency and efficacy of the process.

Topographic ICA (TICA) proposed by Hyvärinen et al. [18] is a modified ICA model, which relaxes the assumption of statistical independency of the components, considering the components topographically closed to each other are not completely independent but have certain dependencies. The dependencies are used to define a topographic order between these components. This provides a very efficient method for separation of the multichannel EEG source signals. Generally, the EEG recordings reveal the sum of the action potentials of the neural cells, which are very complicated to be understood physiologically and mathematically. The dependencies between such sources cannot be simply cancelled out by some statistical assumptions. In this paper, we show how TICA works for the separation of the epileptic seizure EEGs, and how the performance can be improved by introducing novel spacial and frequency constraints in TICA. (In this paper, the constrained TICA is denoted as CTICA).

The paper is organized as follows. Section 2 describes the algorithm development. First, the basic TICA model and principles are explained. Then, the CTICA model is developed. Section 3 gives the experimental results obtained by applying the proposed methods to the epileptic seizure EEGs. The performance of CTICA and TICA is compared, and the superiority of CTICA is demonstrated by comparing with other commonly used ICA algorithms. The final section concludes the paper.

## 2. ALGORITHM DEVELOPMENT

### 2.1. Topographic ICA

The conventional noise-free ICA model can be expressed as

(1) x(t)=As(t),

where **x**(*t*) = [ *x*_1_(*t*), *x*_2_(*t*),…, *x_n_*(*t*)]^*T*^, **x** ∈ ℜ^n^ is the vector of observed signals at time *t*, (⋅)^*T*^ denotes transpose operation, **s**(*t*) = [*s*_1_(*t*), *s*_2_(*t*),…, *s*_m_(*t*)]^*T*^ is the unknown independent source, **s**, ∈ ℜ^*m*^, *m* ≤ *n* for over-determined mixtures, and **A** ∈ ℜ^*n*×*m*^ is the mixing matrix. The estimated sources **y**(*t*) = [*y*_1_(*t*), *y*_2_(*t*),…, *y*_*m*_(*t*)]^*T*^ can be obtained by a separation matrix **W** through the inversion of the above mixing model,

(2) y(t)=Wx(t),

where **W** = **A** ^†^ is the pseudoinverse of the mixing matrix and **WA** = **I**. In the conventional ICA, the sources are assumed to be completely statistically independent, and the estimated signals have no particular order. But in most real applications, some sources may be more or less dependent on each other, such as the EEG sources which are fired from close locations within the cortex. In order to estimate the dependency of the independent components, Hyvärinen et al. proposed the TICA [18]. In TICA, the independency of the components has been relaxed, which means that the sources geometrically far from each other in topography are considered approximately independent and those close to each other are assumed to have certain dependencies. The dependency is defined as the higher-order correlation between the estimated sources, such as the correlation of the energies:

(3) $$\text{cov}(s_{i}^{2},s_{j}^{2})=E\{s_{i}^{2}s_{j}^{2}\}-E\{s_{i}^{2}\}E\{s_{j}^{2}\}\neq 0,$$

where cov (⋅) is the covariance of the two sources *s*_*i*_ and *s*_*j*_, and *E*{⋅} is the expectation operator. Therefore, the estimated sources from the TICA are still uncorrelated, but their energies are not.

In the TICA model, the variances of estimated components are not constant, instead, they are generated by some high-order independent variables. These variables are mixed linearly in the topographic neighborhood, which are defined by a neighborhood function *h*(*i*, *j*). Based on this model, the estimated components in the same neighborhood are energy-correlated. The approximation of the density of source *s* is given as [18]

(4) $$\tilde{p}(s)=\prod_{k}\exp(G(\sum_{i}h(i,k)s_{i}^{2})),$$

where *k* is the index of the components within the same neighborhood. *G*(⋅) is the scalar function defined by incorporating certain nonlinearity. In this work, *G*(⋅) has been defined in [18]:

(5) $$G(y)=-\alpha \sqrt{\varepsilon +y},$$

where *α* and *ε* are scalar constants.

The approximation of the log likelihood of this model is given in the following equation; more details of the derivation can be found in [18]:

(6) $$\log\;\tilde{L}\left(W\right)=\sum_{t=1}^{N}\;\sum_{j=1}^{n}G\left(\sum_{i=1}^{n}h\left(i,j\right){\left(w_{i}^{T}x\left(t\right)\right)}^{2}\right)+N\;\log\left(\left|\det\;W\right|\right),$$

where **w**_*i*_ is the column vector of the unmixing matrix, *N* is the length of the data, and *h*(*i*, *j*) is the neighborhood function, which can be defined as a monotonically decreasing function of some distance. The second term of the above equation can be ignored, since the unmixing matrix is constrained to be orthogonal and the determinant of an orthogonal matrix is one. Therefore, the estimation of the TICA model changes to choosing the optimal matrix **W**_opt_ that maximizes the above log-likelihood function. The estimation of maximization of the log likelihood of (6) can be found by

(7) $$\frac{\partial }{\partial W}\log\;\tilde{L}\left(W\right)|\text{w}={\text{w}}_{\text{opt}}=0.$$

The gradient is obtained as [18]

(8) $$\nabla_{{\text{W}}_{k}}=2\overset{N}{\underset{t=1}{\sum }}x(t)(w_{k}^{T}x(t))\overset{n}{\underset{j=1}{\sum }}h(k,j)g(\overset{n}{\underset{i=1}{\sum }}h(i,j)(w_{i}^{T}x(t{))}^{2}),$$

where *g* (⋅) is the derivative of the scalar function *G*(⋅).

### 2.2. Constrained topographic ICA

The estimated components from the TICA may be dependent if they fall into the same neighborhood, that is, the sources coming from the nearby location will be grouped together. However, the performance of TICA algorithm has certain limits. It may not be easy to get the sources grouped together unless the nearby sources are active at the same time. In [18], in order to obtain better visualization results, the experiment was designed to generate some typical high energy sources, such as biting teeth for 20 seconds. However, in most cases of real applications, the source signals may not be so significant, or there may be only one or two of active sources. Another factor is the number of input channels. It is obvious that the more input channels, the more information one can have and the better results can be achieved. This can be another limitation for the practical applications. However, the performance can be improved by introducing certain constraints into the algorithm.

Adding prior information, as a constraint, to classic ICA has been previously applied to EEG signal separation and analysis [15, 19–23]. The conventional ICA does not exploit the dependency of the sources, therefore, does not always provide the desired outputs. For EEGs, there is valuable prior knowledge which can help to separate the desired sources. In this study, we consider two constraints which are based on spatial and frequency information. Firstly, in the focal epileptic seizures, the location of the seizure sources, “epileptogenic zone,” is often known as the prior information. Secondly, the seizure signals manifest themselves within certain frequency band. Based on the research findings from the clinicians and the neurologists, although the dominant frequency may vary for different types of seizures, the frequency band of the epileptic seizure onset is normally from 2.5 to 15.5 Hz. (Frequencies below 2.5 Hz are considered to be mainly due to eye-blinking artifacts) [24–26]. Therefore, the constraint can be determined based on both spatial and frequency domain information. The model of the constrained TICA problem can be expressed as

(9) $$\max\;J_{m}(W),\;\;\text{s}\text{.t}\text{.}\min\;J_{c}(W),$$

where *J*_*m*_(**W**) is the main cost function, which is based on TICA as shown in (6). *J*_*c*_(**W**) is the constraint which can be defined as minimizing the distance between the output and a reference signal:

(10) $$J_{c}(W)=\arg\;\underset{w}{\min}\overset{N}{\underset{t=1}{\sum }}∥w_{i}^{T}x(t)-y_{r}(t){∥}_{2}^{2},$$

where **y** *_r_* is the reference signal defined based on the spatial and frequency constraints and ‖ ⋅ ‖_2_ measures the Euclidean distance. The CTICA is then changed to an unconstrained function by using a Lagrange multiplier. Therefore, the overall cost function is written as

(11) $$J(W,\text{Λ})=J_{m}(W)-\Lambda J_{c}(W),$$

where Λ = diag{Λ_*ii*_}, *i* = 1 ,…, *m*, is a diagonal weight matrix formed by

(12) $$\text{Λ}=p\cdot \text{diag}(\text{cor}(y_{r},y_{i})),$$

where *p* is an adjust constant, cor(⋅) is the correlation measurement, and *y*_*i*_ is the *i*th estimated source. Then, the update equation is obtained as

(13) $$W(k+1)=W(k)+\mu (k)\{\frac{\partial J_{m}(W)}{\partial W}+\text{Λ}(x{\left(\mathrm{WX}-Y_{r}\right)}^{T})\},$$

where *μ* is the learning rate which is updated iteratively. **Y** *_r_* is the matrixwith the reference signal *y_r_* in each row.

## 3. EXPERIMENT

The experiments consist of the application of the proposed CTICA algorithm to two patients with focal epileptic seizure. Generalized seizure was not considered in this work because the main purpose of this study was to investigate the predictability of epileptic seizure which is possible for only focal seizures. The epileptogenic zone was confirmed by the clinical experts as the prior information. Both patients' data contained epileptic seizure onset were truncated from the original long recording EEGs and were used in the experiments to validate the algorithm. The first experiment compared the performance of CTICA and TICA in terms of the signal-to-interference ratio (SIR). The second experiment provided the comparison of CTICA and three algorithms in terms of correlation measurement. Both experiments used topography to assist the visualization of the results.

In order to evaluate the performance, SIR was defined to be the averaged signal energy for the estimated source *y*(*t*) from the direct source divided by the energy stemming from the other sources; higher value of SIR indicates a better performance:

(14) $$\text{SIR}=\frac{(1/m)\sum_{i}^{m}|W_{ii}^{-1}{|}^{2}<|y_{i}{|}^{2}>}{(1/m(m-1))\sum_{i\neq j}^{m}\sum_{j}^{m}|W_{ij}^{-1}{|}^{2}<|y_{j}{|}^{2}>},$$

where **W**_ii_^−1^ includes the diagonal elements in the inverse of unmixing matrix, that is, the weights from source *y*_i_ to sensor *x*_i_. The off-diagonal elements **W**_ij_^−1^ provide the weights from the source *y*_i_ to the sensor *x*_i_. It shows how the source *y*_i_ interferes the source *y*_i_, since each column of the inverse of unmixing matrix indicates the distribution of each source in the mixtures.

The parameters used in the experiments were set up as follows. In (5), the scalar function *G*(⋅) parameters *α* and *ε* are chosen, respectively, as 1 and 0.005 refering to [18]. The adjust constant *p* in (12) was chosen between 6 to 10 based on the experiments performance. The initial value of learning rate *μ* in (13) was set to 0.1.

### 3.1. Experiment I

#### 3.1.1. Data acquisition and the experiment setup

**Figure 1:**
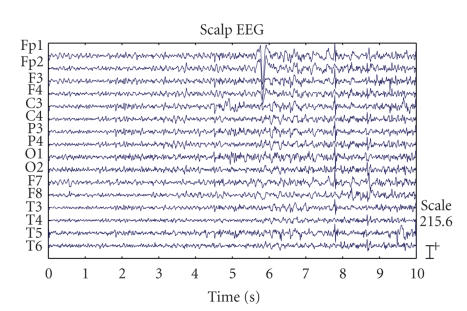
Multichannel EEG signals from an epilepsy patient including the seizure onset.

The multichannel EEGs with the frontal focal epileptic seizure were recorded using the standard silver cup electrodes applied according to the “Maudsley” electrode placement system, which is a modification of the extended 10–20 system [27]. This system provides a more extensive coverage of the lower part of the cerebral convexity, increasing the sensitivity for the recording from basal subtemporal structures. The 16 channels EEGs were sampled at 200 Hz and bandpass filtered in the frequency range of 0.3–70 Hz. The system input range was 2 mV and the data were digitized with a 12-bit analog-to-digital converter [15]. The signals were preprocessed by first removing the baseline to alleviate the effect of low frequency artifacts. Then, the EEGs were filtered by a 10th order Butterworth digital filter with a cut frequency of 45 Hz in order to eliminate the 50 Hz frequency component. The EEGs used in the following experiment were truncated from the original recordings to include the duration of 10 seconds with seizure onset as shown in Figure 1.

#### 3.1.2. Reference

The reference signal was obtained by first averaging the special channels closed to the epileptogenic zone. In these experiments, F3, F4, F7, F8, C3, and C4 were selected. Then, 3–15 Hz bandpass filtering was undertaken to extract the information within the seizure frequency band. The final reference is a vector bounded within the designed spatial and frequency information of the seizure.

#### 3.1.3. Neighborhood function

The neighborhood function indicates how the estimated sources are energy correlated with each other, which can be defined as a function of the width of the neighborhood. In this study, because of the limited number of input channels, the function was chosen as the simple one-dimensional form, such as *h*(*i*, *j*) = 1, if |*i* − *j*| ≤ *m*, otherwise, *h*(*i*, *j*) = 0, where *m* is the width of the neighborhood. It can be noticed that the neighborhood function is symmetric as *h*(*i*, *j*) = *h*(*i*, *j*).

#### 3.1.4. Results

**Figure 2:**
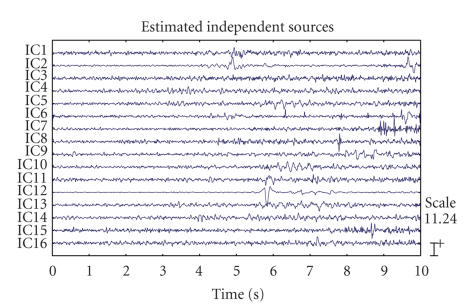
The EEG source signals estimated by TICA.

**Figure 3:**
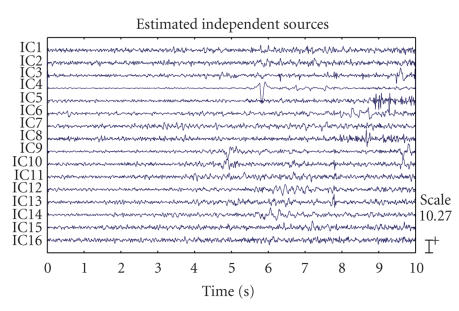
The EEG source signals estimated by TICA.

The separation results of TICA and CTICA are given in Figures 2 and 3. Figure 7 gives the convergence curve of CTICA. Both algorithms used the width of neighborhood *m* = 1. A simple detection rule based on the dominant frequency and respective estimated spectrum is applied to select the sources which have the significant ictal activities. The source with a maximum spectrum amplitude higher than a threshold and also with the dominant frequency in the seizure band, is taken as a seizure source. These sources are IC7, IC8, IC9, and IC10 in Figure 2, IC5, IC6, IC7, and IC8 in Figure 3. One can see that the high amplitude spike signals are separated from the other sources. Another distinct source related to the eye blink can be seen from two of the outputs, which is IC12 in Figure 2 and IC4 in Figure 3.

**Figure 4:**
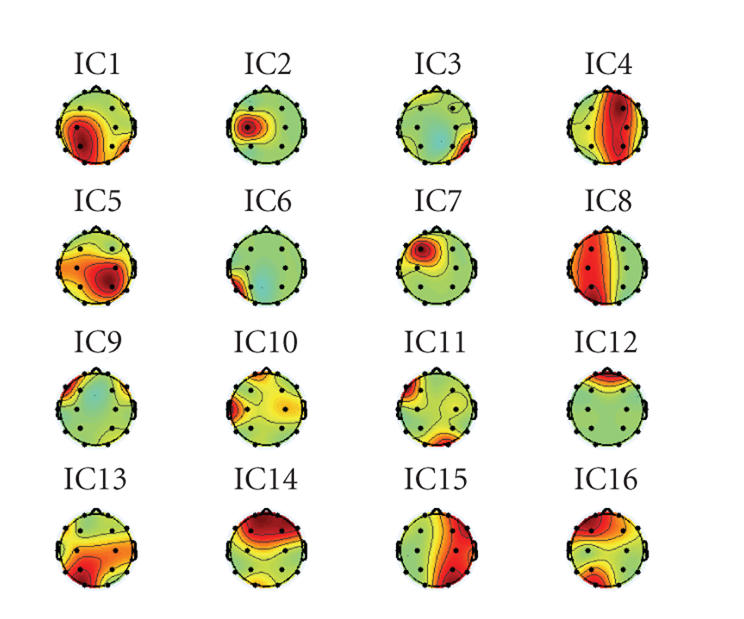
Topography of the estimated EEG sources from TICA.

**Figure 5:**
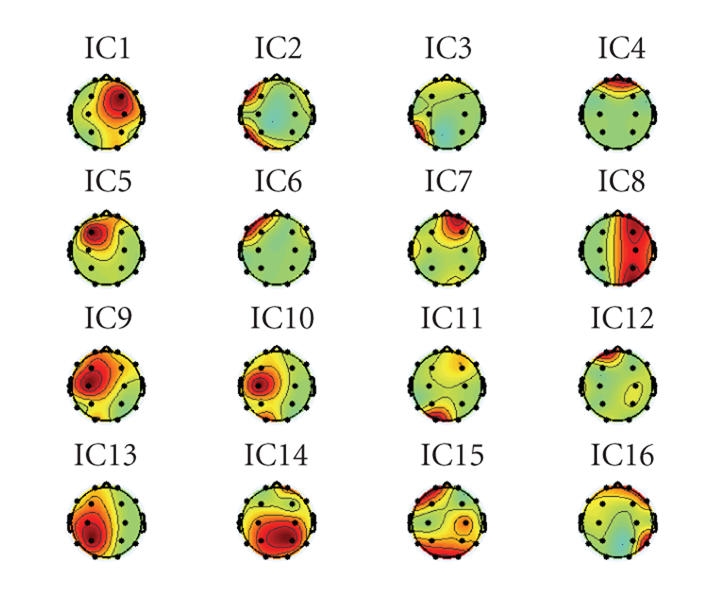
Topography of the estimated EEG sources from CTICA.

It may not be easy to decern the differences between the source candidates only by visual inspection of the time course of the sources, hence the topography was used to help visualization of the results. Figures 4 and 5 provide the topographies of the sources estimated, respectively, by TICA and CTICA. A topography can be obtained by back-projecting the estimated source onto the original signal space, that is, multiplying the column vector of the inverse of unmixing matrix by the corresponding estimated source. Topography reveals how the source signal contributes to each recordings, for example, one can notice that, in both sets of results, the distribution of eye blink (IC12 in Figure 4 and IC4 in Figure 5) appears on the area near the electrodes Fp1 and Fp2. It can be found that the four selected ICs are grouped together. The difference is, in Figure 5, the selected ICs (IC5, IC6, IC7, and IC8) from the CTICA are localized in the frontal region, but in Figure 4, the distribution of the corresponding sources (IC7, IC8, IC9, and IC10) by the TICA are rather dispersed. For instance, for IC10, the spatial distribution is highlighted in both frontal and temporal areas. A similar result can be noticed for IC11.

**Figure 6:**
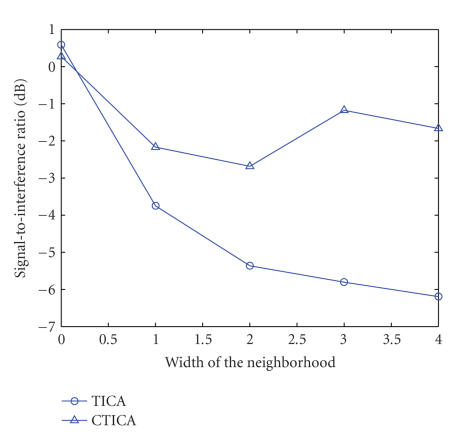
Performance comparison of TICA and CTICA.

**Figure 7:**
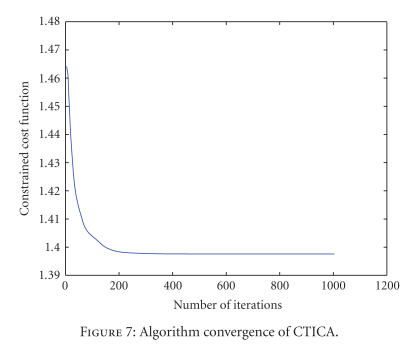
Algorithm convergence of CTICA.

The performance of the algorithm was evaluated by the average of five trials for both TICA and CTICA. The SIR was calculated based on the definition given in (14). Figure 6 illustrates the separation performance (SIR) via the changes of the width of the neighborhood. It can be noticed that the SIR of TICA decreases with the increase of the neighborhood width. This is because the wider the neighborhood is, the more the source will be separated based on energy correlation. However, for the CTICA, due to the spatial and frequency constraints, the SIR slightly decreases at the beginning, then stays approximately at certain level. It shows that, generally, the CTICA has a better performance than the TICA. It also works better than the TICA when the width of the neighborhood increases.

### 3.2. Experiment II

#### 3.2.1. Data acquisition and the experiment setup

In order to validate the performance of CTICA, in the second experiment, CTICA and other three popular ICA algorithms (JADE, SOBI, and Infomax) were applied to a patient with the right temporal seizure. The multichannel EEGs were obtained from a simultaneous EEG-fMRI recording system, in which the data were recorded during the fMRI scanning process. The fMRI scan period was 3 seconds and the scanner artifacts within EEGs were removed by the data provider. The 64 channels EEGs were sampled at 250 Hz and filtered by a 10th order Butterworth low-pass digital filter with a cut frequency of 45 Hz. The data were then truncated with duration of 10 seconds for the separation. The reference signal was formed by averaging the signals from two electrodes T8 and P8. The width of the neighborhood function was *m* = 1. The rest of the parameters was set as in the first experiment.

#### 3.2.2. Results

The performance of the four algorithms were compared in terms of correlation coefficient. For each algorithm, the source which had the maximum correlation with the reference was selected, are the correlation coefficient is shown in Table 1. It can be seen that the source obtained from CTICA has the maximum correlation with the reference and the source from Infomax has the minimum correlation.

**Figure 8:**
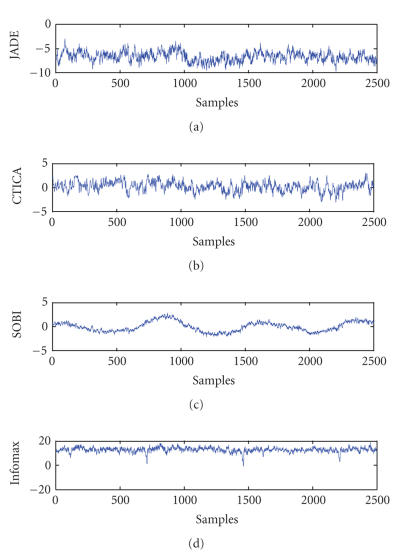
The separated sources from four ICA algorithms. The source which had the maximum correlation with the reference was selected from each algorithm.

The time course of the selected source is shown in Figure 8. It can be noticed that the source from Infomax has clear spikes with a period of 3 seconds, which is the same as the fMRI scan period. The spikes were most likely the fMRI scanner artifacts remained in the EEGs, and Infomax seemed not to separate these artifacts from the desired sources.

**Figure 9:**
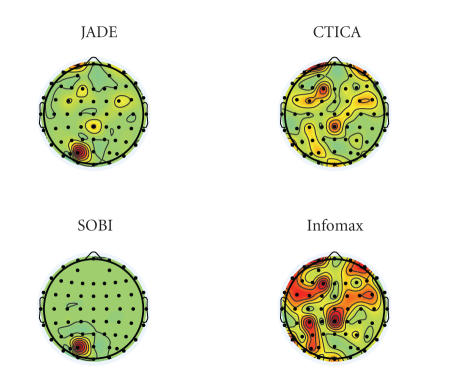
Topography of the selected sources from four algorithms.

Figure 9 compares the topography of the sources selected from the four algorithms. It can be seen that although the topography does not highlight them at the area of interest (which can be due to the depth of the sources), the sources from JADE and CTICA have shown the distribution around the these regions (the right temporal area), and CTICA performs better than JADE. SOBI does not provide the promising result in the area of interest. Topography of the source from Infomax highlights a quite large area in the brain, which is typically caused by the scanning process. This also matches its source time course, in which the spikes were due to the scanner artifacts (as in Figure 8).

## 4. CONCLUSION

A novel constrained topographic ICA algorithm has been developed for separation of the epileptic seizure signals, which not only relaxes the independence assumption of nearby sources, but also further constrains the mixing model in spatial and frequency domains by using application-specific knowledges of epileptic seizures in the form of an averaged and band-limited reference signal. The CTICA algorithm achieves better performance than other ICA algorithms in terms of the SIR and correlation with the reference signal. This provides very promising results for further application of epileptic seizure analysis.

## Figures and Tables

**Table 1:** Correlation between reference and selected source.

JADE	CTICA	SOBI	Infomax
0.5510	0.6832	0.5142	0.3292
